# Preparation, characterization and in vitro–in vivo evaluation of bortezomib supermolecular aggregation nanovehicles

**DOI:** 10.1186/s12951-020-00612-7

**Published:** 2020-04-03

**Authors:** Ming-yue Chen, Ze-kuan Xiao, Xue-ping Lei, Jie-xia Li, Xi-yong Yu, Jian-ye Zhang, Guo-dong Ye, Yu-juan Guo, Guangquan Mo, Chu-wen Li, Yu Zhang, Ling-min Zhang, Zhi-qiang Lin, Ji-jun Fu

**Affiliations:** 1 The Fifth Affiliated Hospital of Guangzhou Medical University, Guangzhou Medical University, Guangzhou, 510700 Guangdong China; 2 The State Key Laboratory of Respiratory Disease, Guangzhou Medical University, Guangzhou, Guangdong China; 3 The Key Laboratory of Molecular Target & Clinical Pharmacology, School of Pharmaceutical Sciences, Guangzhou Medical University, Guangzhou, 511436 China; 4 Institute of Systems Biomedicine, Beijing Key Laboratory of Tumor Systems Biology, School of Basic Medical Sciences, Peking University Health Science Center, Beijing, 100191 China

**Keywords:** Supermolecular nanovehicles, Bortezomib (BTZ), Intermolecular interactions

## Abstract

**Backgrounds:**

Intolerable toxicity and unsatisfactory therapeutic effects are still big problems retarding the use of chemotherapy against cancer. Nano-drug delivery system promised a lot in increasing the patients’ compliance and therapeutic efficacy. As a unique nano-carrier, supermolecular aggregation nanovehicle has attracted increasing interests due to the following advantages: announcing drug loading efficacy, pronouncing in vivo performance and simplified production process.

**Methods:**

In this study, the supermolecular aggregation nanovehicle of bortezomib (BTZ) was prepared to treat breast cancer.

**Results:**

Although many supermolecular nanovehicles are inclined to disintegrate due to the weak intermolecular interactions among the components, the BTZ supermolecules are satisfying stable. To shed light on the reasons behind this, the forces driving the formation of the nanovehicles were detailed investigated. In other words, the interactions among BTZ and other two components were studied to characterize the nanovehicles and ensure its stability.

**Conclusions:**

Due to the promising tumor targeting ability of the BTZ nanovehicles, the supermolecule displayed promising tumor curing effects and negligible systemic toxicity.

## Background

The supermolecule chemistry, which focuses on the weak intermolecular interactions, has been developing quickly these years. As one important branch, the supermolecular nanomedicines has also gained increasing interest. Different from the traditional nanoparticles, the weak intermolecular interactions between the drug and other components produced the supermolecular nano-structures. For example, the intermolecular interactions between the photosensitizer and the short peptides promoted the formation of nanoparticles to play the photodynamic therapeutic effects against cancer [1, 2].

The natural polyphenols, common food ingredients which are extracted from plants such as the green tea, are under extensive investigations now. For example, the gallic acid were used to prepare iron nanoparticles to play the photothermal therapeutic effects against cancer [3]. The epigallocatechin gallate (EGCG) from green tea was used to construct the Sm^III^-EGCG nanocomplexes [4]. Tannic acid (TA) was also employed to cross-link and dope soft conducting polymer hydrogels for spinal cord injury repair [5]. With the help of tannic acid, a tough, self-healing and self-adhesive ionic gel was prepared [6]. All the above applications of polyphenols were based on the formation of coordination bonds between polyphenols and metal ions. Actually, the phenolic hydroxyl groups make polyphenols easy to interact with other molecules and, finally to shape supermolecules. The polyphenols, such as tannic acid, EGCG, and catechin (CAT) were reported to form supermolecular nanovehicles with poloxamer or polyethylene glycol through the hydrogen bonding interactions [7]. However, the nanovehicles were easy to be destroyed by ions and, could not be used for intravenous administration.

Bortezomib (BTZ) is a typical proteasome inhibitor and is a widely used anti-tumor drug. However, the strong cytotoxicity of this drug also leads to the serious compliance problem due to severe weight loss and so on. Nano-drug delivery systems are assumed to be able to reduce the side-effects and improve the therapeutic effects of BTZ. In this work, the authors intended to prepare the BTZ supermolecular nanovehicles. BTZ was reported to form boronic ester between the boron hydroxyl group and the phenolic hydroxyl groups [8, 9]. As a result, BTZ-polyphenol conjugate was first prepared, and then, tried to construct supermolecular nanovehicles later. Specifically, TA was used as it was reported to selectively bind to cancer cells due to the strong interactions between the phenolic hydroxyl groups and the glycoproteins overexpressed on cancer cell membranes [10]. Containing TA in the supermolecular nanovehicles was supposed to enhance the tumor enrichment of the carriers and improve the anti-cancer effects. In addition, TA and poly(*N*-vinylpyrrolidone) (PVP) were reported to form stable nano-structures as the strong interactions between the phenolic hydroxyl groups and the pyrrolidone [11]. As a result, PVP was used to construct supermolecular nanovehicles with the BTZ-TA conjugate. In a word, the supermolecular nano-structures in this work was simply prepared by three components, the active compound BTZ, the polyphenol TA and PVP. Depart from constructing the supermolecules, TA also played the role of active targeting ligand to cancer cells. The supermolecular nanovehicles were assessed in the terms of particle size, the surface charge, the morphology, the storage stability, the drug release, the anti-cancer effects on the cellular level, the distribution in tumor bearing mouse model, the in vivo therapeutic effects and the biocompatibility. Specifically, the supermolecule formation mechanisms were detailed investigated.

## Methods

### Materials

BTZ was bought from Dalian Meilun Biotechnology Company (Dalian, China). TA (M.W. 1700), PVP (M.W. 14,000), coumarin 6, the near-infrared fluorescence dye DiR, MTT were bought from Aladin (Shanghai, China). The Calcein/PI kit and the GreenNuc kit were obtained from Beyotime (Shanghai, China). The HRP labelled anti-Ki67 and anti-PCNA antibodies were bought from Servicebio (Wuhan, China).

### Preparation and characterization of the supermolecular nanovehicles BTZ-NP

5.4 mg of BTZ and 4.8 mg of TA were dissolved in 300 µL of dimethylformamide (DMF), 10.8 mg of PVP was dissolved in 3 mL of deionized water. Then the DMF solution was added to the water solution dropwise with stirring. In this process, a clear solution with light-blue opalescence was produced. Then, the mixed solution experienced dialysis (cutting molecular weight 3000 Da) in deionized water or saline for 48 h to remove the organic solvents. The drug concentration was determined by high performance liquid chromatograph (HPLC) as described in "Dissolution test" Section and, the encapsulation efficacy was calculated according to the following equation:

$${\text{Encapsulation efficacy }}\left( \% \right) \, = \, {{{\text{drug concentration}} \times {\text{volume}}} \mathord{\left/ {\vphantom {{{\text{drug concentration}} \times {\text{volume}}} {\text{drug mass added}}}} \right. \kern-0pt} {\text{drug mass added}}} \times 100\%$$

The blank supermolecular nanovehicles was prepared similarly, except without BTZ addition.

Twenty micrograms of coumarin 6 (C6) was dissolved in the DMF solution before adding to the PVP water solution to prepare the green fluorescence dye labelled BTZ-NP.

Two hundred micrograms of DiR was dissolved in the DMF solution before adding to the PVP water solution to prepare the near-infrared fluorescence dye labelled BTZ-NP.

The particle size and the surface zeta potential of the supermolecular nanovehicles BTZ-NP were measured (Malvern).

The morphology of BTZ-NP was characterized by transmission electronic microscope (TEM, 100 kV) and scanning electronic microscope (SEM, 5 kV). The above sample in deionized water was directly used for TEM and, the freeze-dried sample was used for SEM.

To assess the stability of BTZ-NP, the supermolecular nanovehicles in saline was stored at room temperature and, the particle size of the sample was monitored to reflect the storage stability.

### Dissolution test

The drug release of BTZ-NP was tested in phosphate buffer solutions (PBS) with different pH values (pH 7.4, pH 6.5). In details, 0.8 mL of the BTZ-NP (0.675 mg/mL, equivalent to 0.54 mg of BTZ) was added to the dialysis bag (cutting molecular weight 3000 Da). The dialysis bag was immersed in 40 mL PBS. The shaking rate was 100 rpm and, the temperature was 37 °C. At the time points of 1, 2, 3, 4, 6, 8, 10, 12 h, the samples were withdrawn and analyzed by high performance liquid chromatograph (HPLC) to determine the drug concentration. The mobile phase was methanol and water (pH 2.0) (60 : 40), the detection wavelength was 220 nm, the C18 column (5 µm, 25 cm, Xbridge, Waters) was used, the flow rate was 1 mL/min.

### Hemolysis test

The study was approved by the Ethic Committee of Guangzhou Medical University.

One milliliter of rat blood was centrifugated at 800 rpm for 5 min to remove the plasma, then the blood cells were washed by PBS for three times. Then, the blood cells were suspended in 1 mL of PBS. About 100 µL of the blood cells in PBS was added to 900 µL of the BTZ-NP solution with a serial concentrations of 10, 20, 50, 100, 200, 500, 1000 µg/mL, with PBS and deionized water as negative and positive control. The above samples were placed for 24 h at room temperature. Then, centrifugate the samples at 800 rpm for 5 min to separate the cells and the supernatant. The samples were pictured and, 200 µL of the supernatants were withdrawn and the absorbance at 540 nm was determined to calculate the hemolysis rate.

### Structure characterization of the supermolecular nanovehicles by nuclear magnetic resonance (NMR) and fourier transform infrared spectrum (FTIR)

The following samples were prepared. 5 mg of BTZ was dissolved in 0.5 mL of deuterium DMSO, 5 mg of TA was dissolved in 0.5 mL of deuterium DMSO, 10 mg of PVP was dissolved in 0.5 mL of D_2_O, 20 mg of the freeze-dried BTZ-NP powder was nanodispersed in 0.5 mL of D_2_O. The, the NMR spectra of the samples were determined (Bruker).

The BTZ-NP solution was freeze dried to powder, then, the FTIR spectrum of BTZ-NP powder, TA, PVP and BTZ were scanned (Bruker).

### The isothermal titration calorimetry (ITC) experiment

ITC was employed to investigate the interactions between TA and PVP. 15 mg/mL of TA ethanol solution in the titration syringe was added into 10 mg/mL of PVP ethanol solution in the sample cell. The thermodynamic parameters were determined using the independent bind site model (Nano ITC, TA). The following parameters were determined:

n, the number of binding sites every monomer of the substance in the cell;

ΔH, the change in enthalpy (kJ/mol) upon binding;

ΔS, the binding entropy (J/mol·K);

ΔG, the reaction free energy (kJ/mol), which can be calculated according to the Eq. 2

2 $$\Delta {\text{G}}\; = \;\Delta {\text{H}} - \;{\text{T}}\Delta {\text{S}}$$

### Cell uptake

In this work, the mouse breast cancer cell 4T1 was used, the cells were cultured in DMEM media.

To investigate the uptake of the supermolecular nanovehicles by the malignant cells, the following experiment was carried out. 3 × 10^4^ of 4T1 cells were seeded in 24-well plate and, were allowed to grow overnight in 37 °C, 5% CO_2_ oven. Then, the C6 labelled BTZ-NP was added to the cells for 0.5 h or 1 h, the C6 concentration was 50 ng/mL. After washing the cells with PBS for three times, the cell uptake of the BTZ-NP was determined by the reversed fluorescence microscope (DMi8, Leica).

### The antitumor effects on cellular level

1 × 10^4^ of 4T1 cells or L02 cells (human hepatic cell) were seeded in 96-well plate and, were allowed to grow overnight in 37 °C, 5% CO_2_ oven. Then, the BTZ-NP or free BTZ was added to the cells to get a final serial drug concentrations of 10, 20, 50, 100 nM, the PBS treated cells were used as control. After incubating for 24 h, the viability of the cells were assayed by MTT method. Similarly, the blank supermolecular nanovehicles was added to 4T1 cells to make a final concentration equivalent to BTZ of 0, 10, 20, 50, 100, 200, 500, 1000, 2000 nM to assess the influence of TA and PVP.

4T1 cells were seeded in 10-cm plate and, were allowed to grow overnight. Then, the BTZ-NP was added to the cells to make the final BTZ concentration of 20 nM. After incubating for 12 h, the medium was discarded and the cells were washed by cold PBS for three times. Then, the cells were fixed in 2.5% glutaraldehyde solution and analyzed by TEM to see cell apoptosis.

3 × 10^4^ of 4T1 cells were seeded in 24-well plate and, were allowed to grow overnight in 37 °C, 5% CO_2_ oven. Then, the BTZ-NP or free BTZ was added to the cells to make the final BTZ concentration of 20 nM. After incubating for 24 h, the cells were analyzed by Calcein/PI staining.

3 × 10^4^ of 4T1 cells were seeded in 24-well plate and, were allowed to grow overnight in 37 °C, 5% CO_2_ oven. Then, the BTZ-NP or free BTZ was added to the cells to make the final BTZ concentration of 20 nM. After incubating for 24 h, the caspase 3 expression was analyzed by the GreenNuc kit (Beyotime, China).

### Distribution of the supermolecular nanovehicles in tumor bearing mouse model

The animal experiments were approved by the Ethic Committee of Guangzhou Medical University.

In details, 6–8 week old female Balb/c mice were subcutaneously (*s.c.*) injected with 1 million of 4T1 cells in 200 µL of PBS to establish the xenograft tumor mice model. After the tumor reaching 100–200 mm^3^, the BTZ-NP containing 1 µg of the near-infrared dye DiR was intravenously (i.v.) administered to each mouse. Then, the distribution of the BTZ-NP in tumor bearing mice was determined by imaging at time points of 0.5, 2, 4, 6, 8, 10, 12, 24, 28, 32, 36, 48 h. The ex/em wavelengths were 740/790 nm (IVIS Lumina, PerkinElmer).

6–8 week old female Balb/c mice were subcutaneously (s.c.) injected with 1 million of 4T1 cells in 200 µL of PBS to establish the xenograft tumor mice model. After the tumor reaching 200–300 mm^3^, the BTZ-NP or BTZ solution containing 100 µg of the drug was intravenously (i.v.) administered to each mouse. Six hours later, the mice were sacrificed and, the tumor tissue and the main organs of heart, liver, spleen, lung, kidney were weighed and acid digested by concentrated nitric acid of the same volume. After diluting the samples with deionized water to nitric acid concentration of 5%, the concentrations of boron element was determined by ICP-MS.

### Antitumor effects and the biocompatibility of the BTZ-NP

6–8 week old female Balb/c mice were subcutaneously (s.c.) injected with 1 million of 4T1 cells in 200 µL of PBS to establish the xenograft tumor mice model. After the tumor reaching 100–200 mm^3^, the mice were divided into three groups (n = 6), the PBS control group, the BTZ solution group and the BTZ-NP group. The BTZ-NP, the BTZ solution or PBS were i.v. given to the animals every 3 days and, the BTZ dose of the first two groups was 20 µg of BTZ per mouse. The tumor sizes and body weights were measured throughout the experiment and, the tumor volume was calculated according to the equation: volume = length × width^2^/2. At the end of the experiment, the mice were sacrificed and, the blood samples were collected and assayed to reflect the systemic toxicity of the treatments. The tumor tissues and the main organs of heart, liver, spleen, lung, kidney were sliced and H & E stained. The tumor tissues were weighed and pictured. In addition, the active caspase 3, the Ki67 and the PCNA expression were determined by immunohistochemistry (IHC).

### Statistic analysis

All values were expressed as mean ± standard deviation (SD). All comparisons were performed by the two-tailed Student’s t test. A *p* value less than 0.05 was taken as statistically significant and a *p*-value less than 0.01 was considered to be highly significant.

## Results and discussions

### Preparation and characterization of the supermolecular nanovehicles

**Fig. 1:**
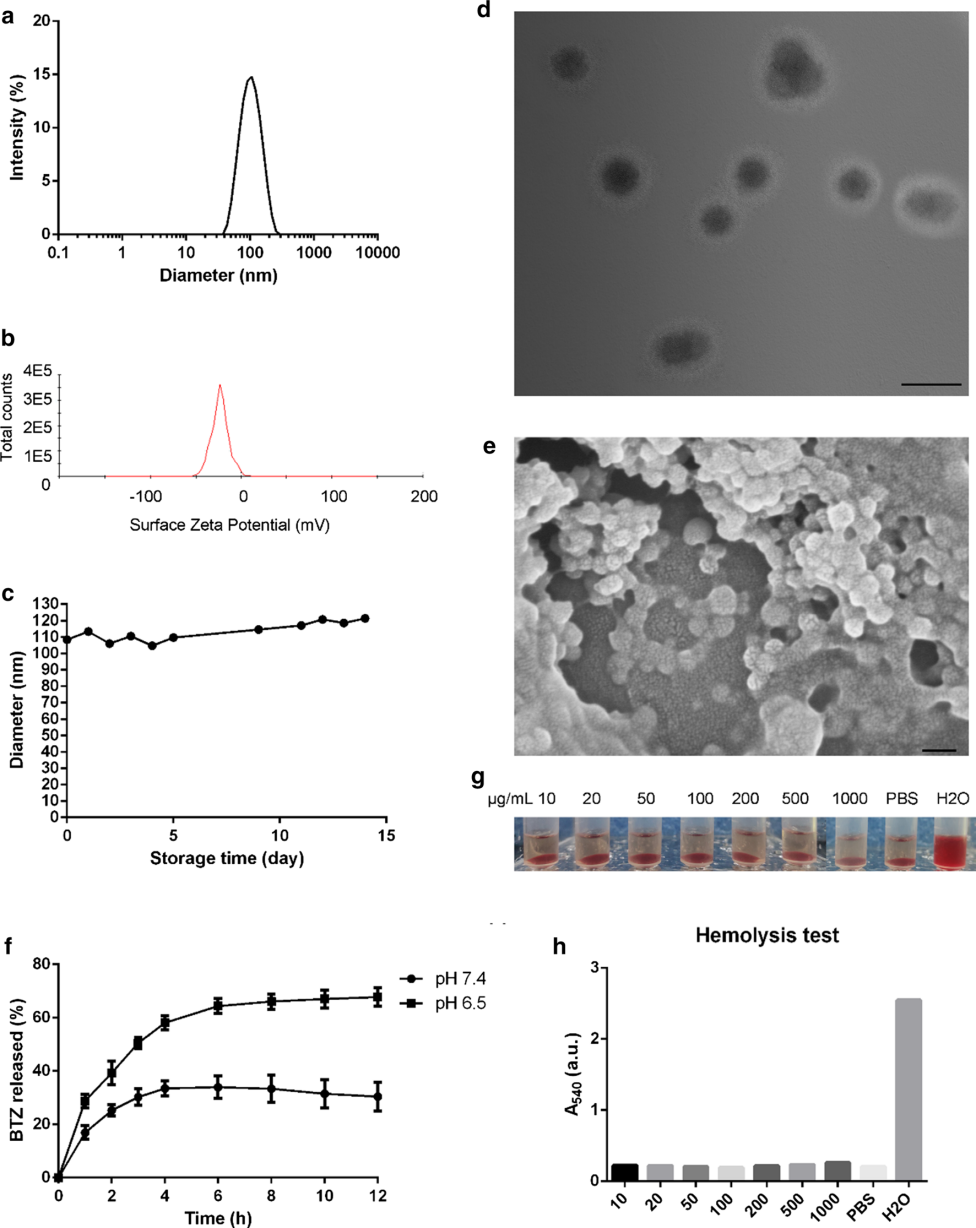
**a** The particle size of BTZ-NP measured by dynamic light scattering method; **b** The surface zeta potential of BTZ-NP; **c** The particle size of BTZ-NP in saline during its storage at room temperature; **d** TEM images of the BTZ-NP (the scale bar represents 100 nm); **e** SEM images of the BTZ-NP (the scale bar represents 100 nm); **f** The drug release profiles of BTZ-NP in phosphate buffer solution; **g** and **h** The hemolysis pictures of BTZ-NP and the quantitative results

The supermolecular nanovehicles BTZ-NP was simply prepared by adding the mixture of BTZ and TA in the DMF solution to the PVP water solution before removing the organic solvent. The supermolecule BTZ-NP exhibited typical light-blue opalescence without detectable granules, which indicated the formation of nanovehicles. The encapsulation efficacy of BTZ was determined to be about 50%. In details, the particle size of BTZ-NP was about 100 ± 40 nm and, the surface zeta potential was about − 20.0 ± 8.5 mV (Fig. 1a, b). The BTZ-NP show spherical morphology in both TEM and SEM images (Fig. 1d, e), with the diameter of about 50−100 nm. As the particle size measured by dynamic light scattering was the hydrodynamic diameter with hydration shell, the prior result was bigger than the latter one. In addition, the TEM image clearly shows that the nanovehicles display core–shell structure. It is anticipated that the hydrophilic TA and PVP constructed the shell and, all the three components formed the core.

Figure 1c shows the storage stability of BTZ-NP in saline at room temperature in the term of particle size. The BTZ-NP exhibited a nearly constant particle size throughout the whole experiment, implying the excellent storage stability in saline. Actually, as the weak intermolecular interactions produced supermolecular structures, the supermolecules are inclined to be destroyed by ions. The phenomenon retards the wide applications of supermolecules. For example, the polyphenols, such as TA, EGCG and CAT were reported to form supermolecular nanovehicles with poloxamer or polyethylene glycol through the hydrogen bonding interactions [7]. However, the nanovehicles were easy to be destroyed by ions and, was not suitable for i.v. administration. However, the BTZ-NP nanovehicles exhibited superior stability in saline, facilitating its application as nano-drug delivery systems.

Figure 1f shows the drug release profiles of BTZ-NP in phosphate buffer solution with different pH values. In the dissolution media of pH 7.4, the active substance released in the first 4 h and, the dissolution profiles reached the plateau from then on, the total drug released was about 35%. In contrast, the drug quickly released in the first 6 h and then slowly released in the dissolution media of pH 6.5. At 12 h, about 70% of the drugs released. In other words, the BTZ-NP displayed a pH-dependent drug release, with faster drug release in slightly acid environment and slower drug release in neutral environment. The pH–sensitive phenylboronate ester account for the pH-dependent drug release. In the acid environment, the phenylboronate ester tends to break and release free BTZ. As tumor tissue is well known for its slightly acid microenvironment, it is assumed that BTZ-NP will display pH-sensitive and tumor-specific drug release.

Concerning the safety of the supermolecular nanovehicles, the hemolysis test was carried out. As shown in Fig. 1g, h, the BTZ-NP did not produce obvious hemolysis even when its concentration increased to 1000 µg/mL. Quantitatively, the hemolysis rates of all the BTZ-NP groups were similar to that of the PBS group, while deionized water resulted in complete hemolysis. The results proved the satisfactory safety of the supermolecular nanovehicles.

### The supermolecule formation mechanisms research

The formation mechanisms of the supermolecular nanovehicles BTZ-NP were investigated by ^1^H-NMR spectra and thermodynamic study.

**Fig. 2:**
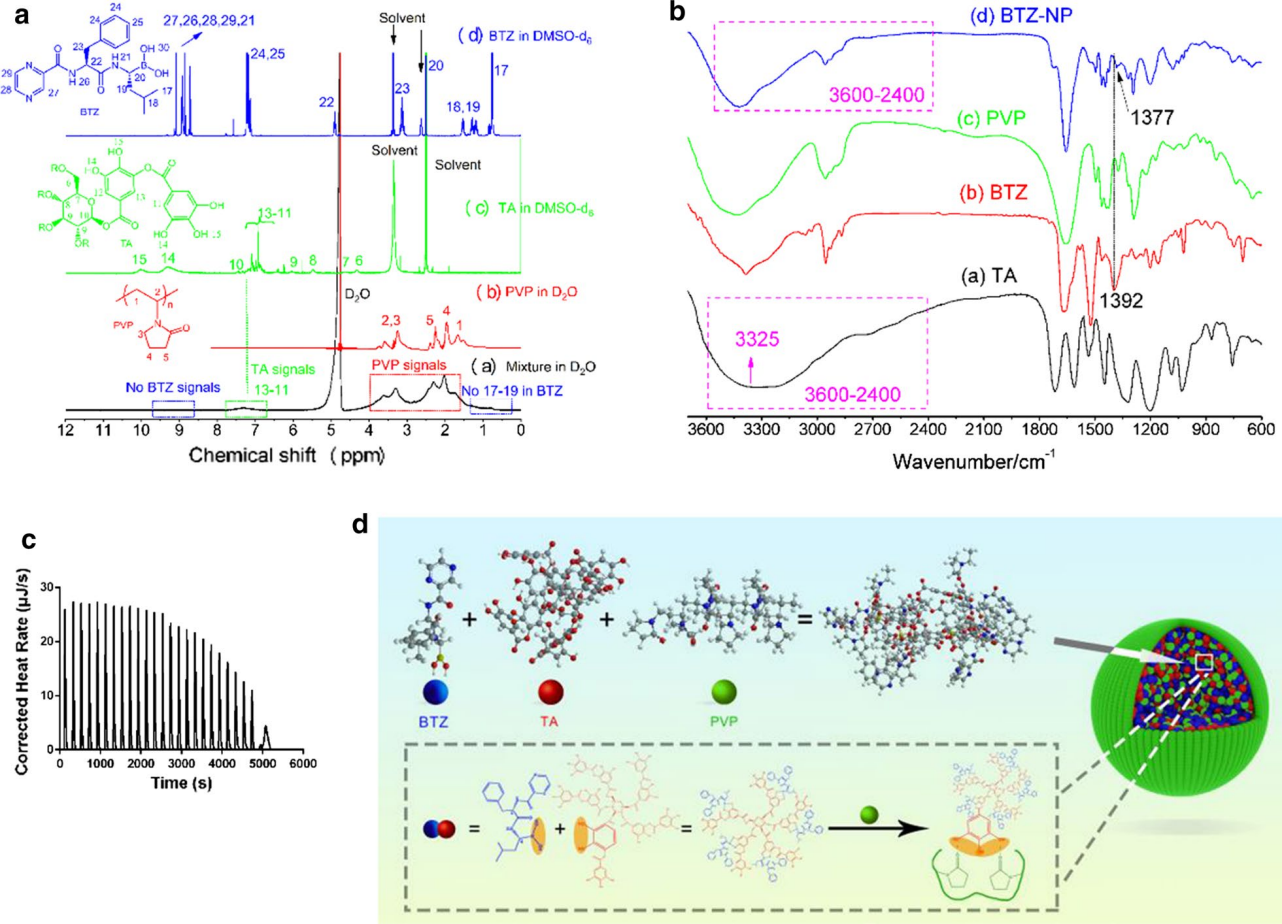
**a**^1^H-NMR spectra of BTZ, TA, PVP and the supermolecule BTZ-NP; **b** FTIR spectra of BTZ, TA, PVP and the supermolecule BTZ-NP; **c** The isothermal titration calorimetry profile of the TA in the titration syringe and the PVP in the sample cell; **d** The structure of the BTZ-NP supermolecular nanovehicles

First, ^1^H-NMR spectra was used to analyze the structure of the supermolecule BTZ-NP. Figure 2a shows that the BTZ in DMSO exhibited typical peaks in the regions of 8–10 ppm (27, 26, 28, 29, 21), 7–8 ppm (24, 25), 5 ppm (22) and other peaks (23, 20, 18, 19, 17). However, in the supermolecular structure of BTZ-NP in D_2_O, none of the BTZ peaks was detectable. It was clear that the peaks corresponding to (27, 26, 28, 29, 21) and (17, 19) were absent in the ^1^H-NMR spectra of BTZ-NP in D_2_O. In contrast, the typical peaks of PVP (2, 3, 5, 4, 1) were clearly observed in the ^1^H-NMR spectra of BTZ-NP in D_2_O and, the TA peaks (13, 12, 11) were also detectable. Moreover, the phenolic hydroxyl groups of TA (15, 14) did not display detectable signals in the ^1^H-NMR spectra of BTZ-NP in D_2_O. The results proposed a special structure of the supermolecular nanovehicles, with BTZ and part of TA constructed the hydrophobic core and, PVP and part of TA shaped the hydrophilic shell. The proposed structure matches the TEM image well. It seems that the TA played the role of linker to bridge the hydrophobic BTZ and the hydrophilic PVP. As discussed in the introduction section, the boron hydroxyl group of the BTZ and the phenolic hydroxyl groups of the TA formed the boron esters, in addition, the phenolic hydroxyl groups of the TA and the carbonyl groups of the PVP formed the hydrogen bonds. In a word, the intermolecular interactions between TA and other two components contributed to the supermolecule formation.

FTIR was also employed to validate the supermolecular structure (Fig. 2b). Firstly, the characteristic O–H stretching mode of the free TA is commonly seen as a broad peak in the range of 2400–3600 cm^−1^ [12, 13], which was also evidenced by the FTIR of the free TA with a peak center at the 3325 cm^−1^. However, this peak was narrowed in the BTZ-NP, indicating that the –OH of the TA was reacted with the phenylboronic acid structure of the BTZ; secondly, the B-O stretching mode of the free BTZ was found at the 1392 cm^−1^, this peak was shifted to 1377 cm^−1^ in the phenylboronate ester for the BTZ-NP, also suggesting that the –OH groups of the free BTZ was reacted with the TA. The shift of this peak to the lower wavenumber was also observed in the previous literature [14]. Hence, the formation of phenylboronate ester between the TA and the BTZ was successfully confirmed by the FTIR.

The ITC was used to investigate the intermolecular interactions between TA and PVP, with TA ethanol solution in the titration syringe and PVP ethanol solution in the sample cell. The ITC profile was shown in Fig. 2c. The thermodynamic parameters of ΔH, ΔS and ΔG were − 100 kJ/mol, − 163.1 J/(mol K) and − 51.37 kJ/mol, respectively. The number of binding sites every monomer of PVP was calculated to be 10. As ΔG < 0, it was assumed that the binding of PVP with TA was autogenic. It is known that the enthalpy change reflects the interaction strength due to the formation of hydrogen bonds as well as vander Waals interactions. The entropy change reflects the hydrophobic interactions between PVP and TA and the conformational change that occurs in the formation of the complex. − T·ΔS > 0 means the structures of TA and PVP switched from disorder to ordered. This point is easy to understand, as it is obvious that the free PVP and TA are unordered and the formed complex are ordered. ΔH < 0 means there are hydrogen bonds and vander Waals interactions between PVP and TA, and the complex formation is driven by enthalpy. In a word, the intermolecular hydrogen bonds between the carbonyl groups of PVP and the phenolic hydroxyl groups of TA in the form of the negative binding enthalpy encouraged the formation of the ordered supermolecular complex BTZ-NP. The thermodynamic research between BTZ and TA was not carried out due to the lack of suitable common solvents.

Concerning the ^1^H-NMR spectra and the ITC results, the structure of the BTZ-NP supermolecular nanovehicles was illustrated in Fig. 2d.

### Cell uptake and the antitumor effects on the cellular level

**Fig. 3:**
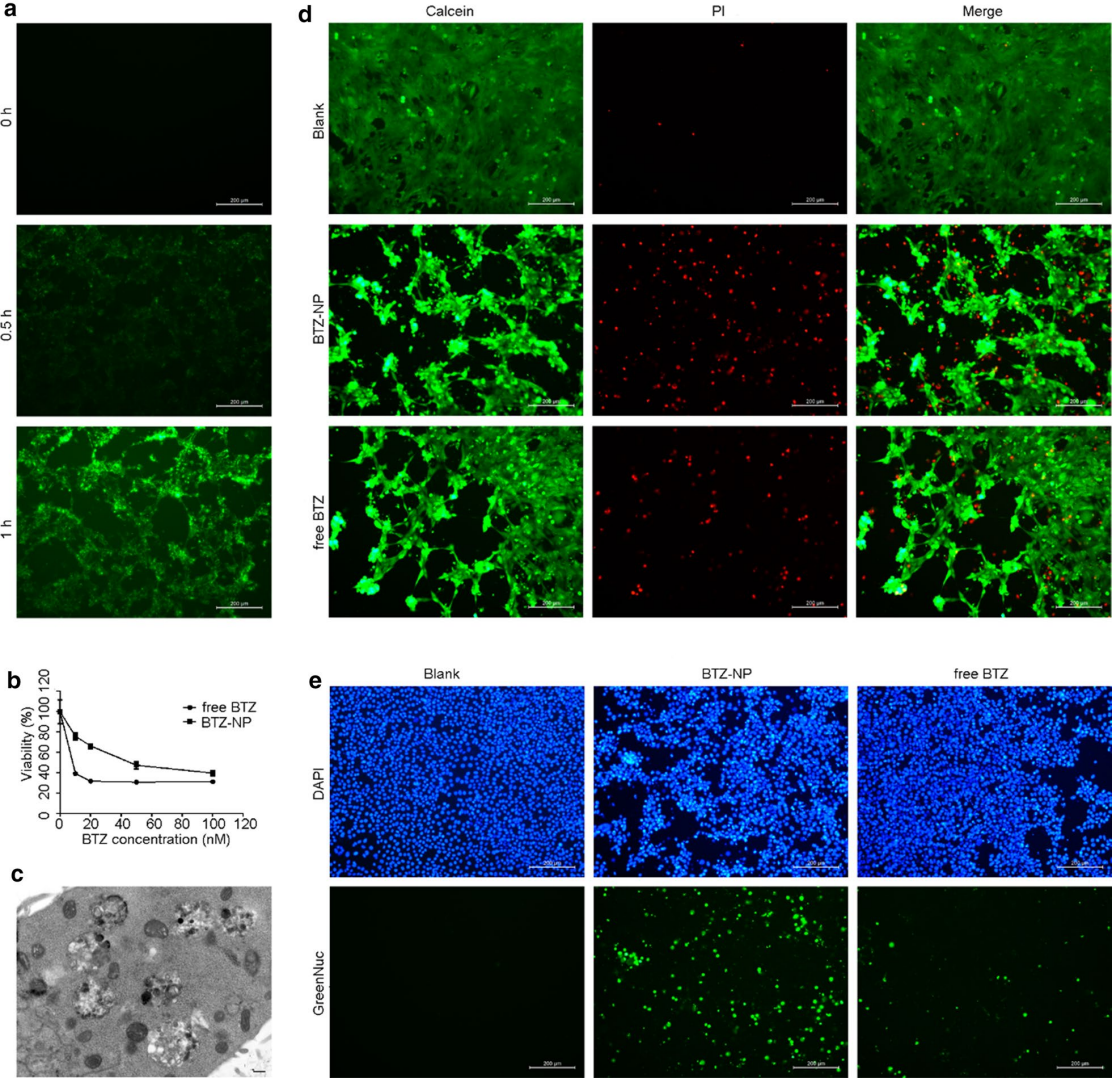
**a** The BTZ-NP uptake by the 4T1 cells; **b** The viability curves of 4T1 cells after incubation with BTZ-NP or free BTZ for 24 h, measured by MTT method; **c** The 4T1 cell apoptosis detected by TEM (the scale bar represents 200 nm); **d** The live/dead assay of 4T1 cells exposed to PBS, BTZ-NP or free BTZ, measured by Calcein/PI; **e** The caspase 3 expression in 4T1 cells exposed to PBS, BTZ-NP or free BTZ, measured by the GreenNuc kit

The BTZ is proteasome inhibitor and, should be taken up by cancer cells to play the therapeutic effects. In this section, the mouse breast cancer cell 4T1 was used to evaluate the cell uptake of BTZ-NP. Figure 3a shows that the BTZ-NP displayed a time-dependent cell uptake, with moderate cell uptake after merely 0.5 h incubation and more obvious cell uptake after 1 h incubation.

Figure 3b shows the antitumor effects of BTZ-NP and free BTZ on 4T1 cells. It was clear that both BTZ-NP and free BTZ exhibited a concentration-dependent cell killing effects, in other words, the antitumor effect increases with increasing BTZ concentrations. It is worth noting that both BTZ-NP and free BTZ played obvious therapeutic effects on the cellular level when the drug concentration was as low as nM. However, Fig. 3b shows that the toxicity of free BTZ was stronger than BTZ-NP, with enhanced cell death rate at each drug concentration in the prior group. Perhaps the drug should release from BTZ-NP before playing the toxic effects, while the free drug directly played the toxic effects, as a result, the apparent toxicity of free BTZ was stronger than BTZ-NP after the “same” exposure time. With the increasing drug concentration, the antitumor effects of BTZ-NP and free BTZ approach, this point further demonstrate the above assumption. Actually, free drugs often exhibited superior performance than nano-carriers on the cellular level as free drugs are more likely to diffuse into cells than nanovehicles [15]. Anyhow, the phenomenon would not bring in too much concerns, as the therapeutic effect was greatly influenced by the drug distribution in vivo, which aspect was not taken into account in this experiment. As the toxicity of BTZ is broad–spectrum, as anticipated, BTZ-NP could not distinguish healthy cells from malignant cells, with detectable toxicity to L02 cells (Additional file 1: Fig. S1).

To assess the influence of TA and PVP, the 4T1 cells were also treated by blank supermolecular nanovehicles without BTZ. Up to the concentration equivalent to 2000 nM of BTZ, there was no detectable toxicity (Additional file 1: Fig. S2). The results indicated the excellent compatibility of the excipients.

4T1 cell apoptosis after BTZ-NP treatment was obviously observed in Fig. 3c. There are many vacuoles in the cell. The vacuoles originate from endoplasmic reticulum in the dead cells and, is typical indicator of cell apoptosis.

As shown in Fig. 3d, the 4T1 cells treated by PBS grew well with negligible cell death. In contrast, the 4T1 cells exposed to 20 nM of BTZ-NP exhibited obvious cell death, with fewer cells survived and significantly more cells died. The 4T1 cells exposed to 20 nM of free BTZ displayed similar trend to the BTZ-NP group. In details, compared to the free BTZ group, the BTZ-NP group seemed to induce more cell death.

Caspase 3 expression is an important symbol of cell apoptosis. The GreenNuc kit was used to detect the caspase 3 expression in this work. The GreenNuc is a short peptide DEVD which is linked to the DNA binding fluorescence dye. The DEVD sequence is highly negative charged and, as a result, inhibits the binding of the fluorescence dye and the nucleus DNA. The active caspase 3 could recognize the DEVD sequence and release the fluorescence dye. The binding of the nucleus DNA and the fluorescence would produce nucleus with green fluorescence, and indicate the expression of active caspase 3. Figure 3e shows that there was no cell apoptosis in the PBS treated 4T1 cells. In contrast, obvious cell apoptosis was observed in the BTZ-NP treated cells. However, the free BTZ treatment resulted in much weaker cell apoptosis signals compared to the BTZ-NP group.

### In vivo distribution of the BTZ-NP

**Fig. 4:**
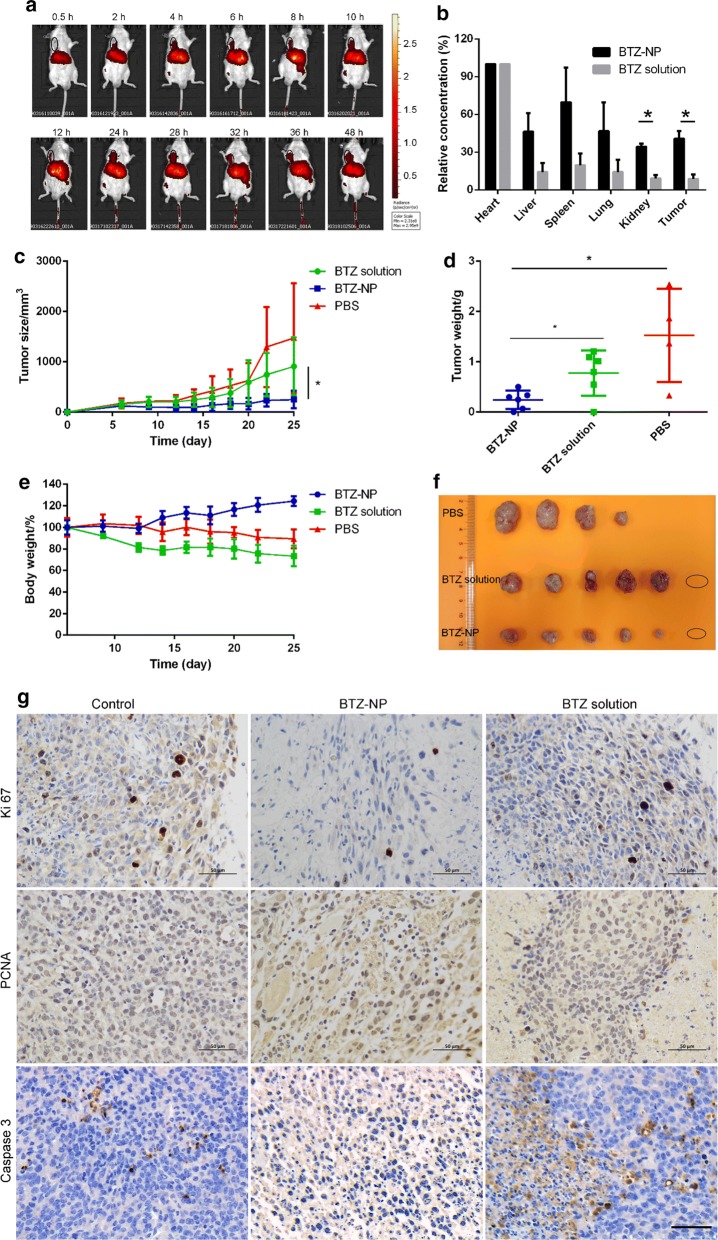
**a** The distribution of DiR labelled BTZ-NP in tumor bearing mouse measured by near-infrared fluorescence imaging (the black circle indicates the tumor tissue); **b** The ICP-MS results of the boron element distribution in the main organs and the tumors (* indicates statistically different, *p* < 0.05); **c** The tumor growth profiles (* indicates statistically different, *p* < 0.05); **d** The statistic results of the tumor weights at the end of the experiment (* indicates statistically different, *p* < 0.05); **e** The body weight curves; **f** The picture of the tumor tissues at the end of the experiment (the circle implies no tumor tissue observed); **g** IHC staining of the tumor tissues (the scale bar represents 50 µm)

The distribution of therapeutic agent in vivo plays an important role in realizing the therapeutic effects as well as bringing in adverse effects. The drug distribution of BTZ-NP was assessed by near-infrared fluorescence imaging. As shown in Fig. 4a, the DiR labelled BTZ-NP first appeared in the liver in the first half an hour after i.v. administration. Two hours later, the BTZ-NP started to enrich in the tumor tissue due to the enhanced permeability and retention (EPR) effects. The fluorescence strength in the tumor site increased in the first 24 h and, reached the maximum in the following 24 h. The selective distribution of the BTZ-NP in the tumor would guarantee promising anticancer effects.

The boron element was measured by ICP-MS to indicate the drug distribution in vivo, as shown in Fig. 4b. The results show that both BTZ-NP and the free BTZ were inclined to accumulate in the heart, with the boron element concentration reaching 162 and 262 ppb in these two groups. The BTZ-NP exhibited decreased drug distribution in the heart than the free BTZ and, this feature would help reducing the cardiotoxicity. The boron element in the tumors of the BTZ-NP and the BTZ solution groups reached 69 and 20 ppb, respectively. Assuming the drug concentration in the heart as 100%, the drug in the tumors of the two groups were 40.7% and 8.8%. The drug distribution in the tumor of the BTZ-NP group was statistically higher than the BTZ solution group. The superior enrichment of the BTZ-NP in the malignant tissue was ascribed to the EPR effects of the nano-drug delivery systems and, would promise profound therapeutic effects [16–24].

### The therapeutic effects and the biocompatibility of the BTZ-NP

The therapeutic effects were evaluated in the aspects of tumor growth profiles, tumor weights, the Ki67 and PCNA expression in the tumor tissues. The biocompatibility was assessed in the aspects of body weight changes, H & E staining of the main organs and the blood analysis.

As shown in Fig. 4c, the PBS treated tumor bearing mice saw a rapid tumor growth, with the tumors reaching about 1500 mm^3^ at the end of the experiment. In contrast, the BTZ solution treatment helped to slow the tumor growth, with the tumors reaching about 900 mm^3^ at day 25. The BTZ-NP treatment produced the most satisfying antitumor effects, with the tumors slowly grew to about 250 mm^3^ at the end of the experiment. Figure 4f shows the tumor samples at the end of the experiment, it was clear that the PBS treatment resulted in the biggest tumors and, both BTZ solution and BTZ-NP treatment produced smaller tumors. In details, the BTZ solution treatment produced moderate tumor growth inhibition and, the BTZ-NP treatment produced strong tumor growth inhibition. It is worth noting that both BTZ groups saw a complete tumor regression in one mouse. The average tumor weights of the BTZ-NP group, the BTZ solution group and the PBS group were 0.24 g, 0.78 g and 1.53 g, respectively (Fig. 4d). The tumor weights of the BTZ-NP group and the BTZ solution group were statistically different (*p* < 0.05).

Figure 4e shows the body weight changes of the three groups. The PBS group exhibited a slight body weight loss of about 10% and, the BTZ solution group displayed an obvious body weight loss of more than 20%. In contrast, the BTZ-NP group shows a weight gain of about 20%. The results implied that the supermolecular nanovehicles greatly reduced the adverse effects of BTZ and improved the patients’ compliance.

The cell apoptosis in the tumor tissues was evaluated. The caspase 3 activation is an important indicator of cell apoptosis. As shown in Fig. 4g, there was negligible caspase 3 activation in the tumors of the control group, while caspase 3 activation was greatly improved in the BTZ solution and BTZ-NP group, especially in the latter one. The Ki67 and PCNA expressions in the tumor tissues were measured to evaluate the cell proliferation. The PBS group shows strong Ki67 and PCNA expression in the tumors, the BTZ solution treatment reduced the Ki67 and PCNA expression and, the expression of the proliferation markers was further reduced in the BTZ-NP group. All the above results indicated that the BTZ-NP greatly inhibited cancer cell proliferation and induced cancer cell apoptosis.

Additional file 1: Fig. S3 shows the H & E staining of the tumor tissues and the main organs. The main organs of heart, liver, spleen, lung and kidney in all the three groups displayed the normal morphology, without obvious pathological changes. The results indicated that the BTZ solution and the BTZ-NP treatments did not produce long-term toxicity. In addition, the tumor tissue of the PBS group and the BTZ solution group exhibited healthy cell morphology, while the BTZ-NP group displayed obvious necrosis areas in the tumors. The above phenomenon indicated that the therapeutic effects of the BTZ-NP was superior to the BTZ solution.

The blood analysis results shown in Additional file 1: Fig. S4 further illustrated that the BTZ solution and BTZ-NP treatments did not bring in long-term toxicity, as there was not statistic changes in the blood cells (*p* > 0.05).

## Conclusions

In this work, the supermolecular nanomedicine BTZ-NP was simply prepared with other two components, the PVP and the natural polyphenol TA. The BTZ-NP was negative charged and displayed stable nano-structure in saline. The BTZ-NP shows pH-dependent and tumor-microenvironment sensitive drug release. The supermolecular nanovehicles BTZ-NP was constructed by the boronic ester between the boron hydroxyl group of the BTZ and the phenolic hydroxyl groups of the TA, as well as the intermolecular interactions between the TA and the PVP. The BTZ-NP displayed satisfying 4T1 breast cancer cell uptake and comparable toxicity to the free BTZ against 4T1 cells on the cellular level. In addition, the BTZ-NP displayed enhanced caspase 3 activation in 4T1 cells than the free BTZ. The near-infrared fluorescence experiment shows that the BTZ-NP specifically enriched in the tumor tissue. The quantitative ICP-MS determination further validated that the BTZ-NP distributed in the tumor was statically higher than the free BTZ. The BTZ-NP shows superior therapeutic effects in retarding tumor growth, inducing cell apoptosis and necrosis, as well as reducing cell proliferation in the tumor tissue than the BTZ solution. Both the BTZ-NP and the BTZ solution show tolerable systemic toxicity in the terms of tissue morphology of the main organs and the routine blood test. However, the BTZ solution treated mice show weight loss while the BTZ-NP treated mice show weight gain, this phenomenon indicated the reduced systemic toxicity of the BTZ-NP.

## Supplementary information

**Additional file 1: Fig. S1.** The survival rate of L02 cells treated by BTZ-NP. **Fig. S2**. The survival rate of 4T1 cells exposed to blank supermolecular nanovehicles without BTZ. **Fig. S3.** H & E staining of the tumor tissues and the main organs of heart, liver, spleen, lung, kidney. **Fig. S4.** The blood analysis results of the tumor bearing mice after receiving different treatments.

## Data Availability

All data used to generate these results is available in the main text.

## Abbreviations

BTZ: Bortezomib
BTZ-NP: Bortezomib supermolecular nanovehicles
PVP: Poly(*N*-vinylpyrrolidone)
C6: Coumarin 6
SEM: Scanning electronic microscope
TEM: Transmission electron microscope
NMR: Nuclear magnetic resonance
FTIR: Fourier transform infrared spectrum
ITC: Isothermal titration calorimetry
