# Supplementary material for: Preparation, characterization and in vitro–in vivo evaluation of bortezomib supermolecular aggregation nanovehicles
Source: J Nanobiotechnology. 2020 Apr 3;18:57. doi: 10.1186/s12951-020-00612-7 (PMC7118915; doi:10.1186/s12951-020-00612-7)
Supplement: Supplementary file 1 — Additional file 1: Fig. S1. The survival rate of L02 cells treated by BTZ-NP. Fig. S2. The survival rate of 4T1 cells exposed to blank supermolecular nanovehicles without BTZ. Fig. S3. H & E staining of the tumor tissues and the main organs of heart, liver, spleen, lung, kidney. Fig. S4. The blood analysis results of the tumor bearing mice after receiving different treatments. [file 12951_2020_612_MOESM1_ESM.docx]

Fig. S1 The survival rate of L02 cells treated by BTZ-NP.





Fig. S2 The survival rate of 4T1 cells exposed to blank supermolecular nanovehicles without BTZ.


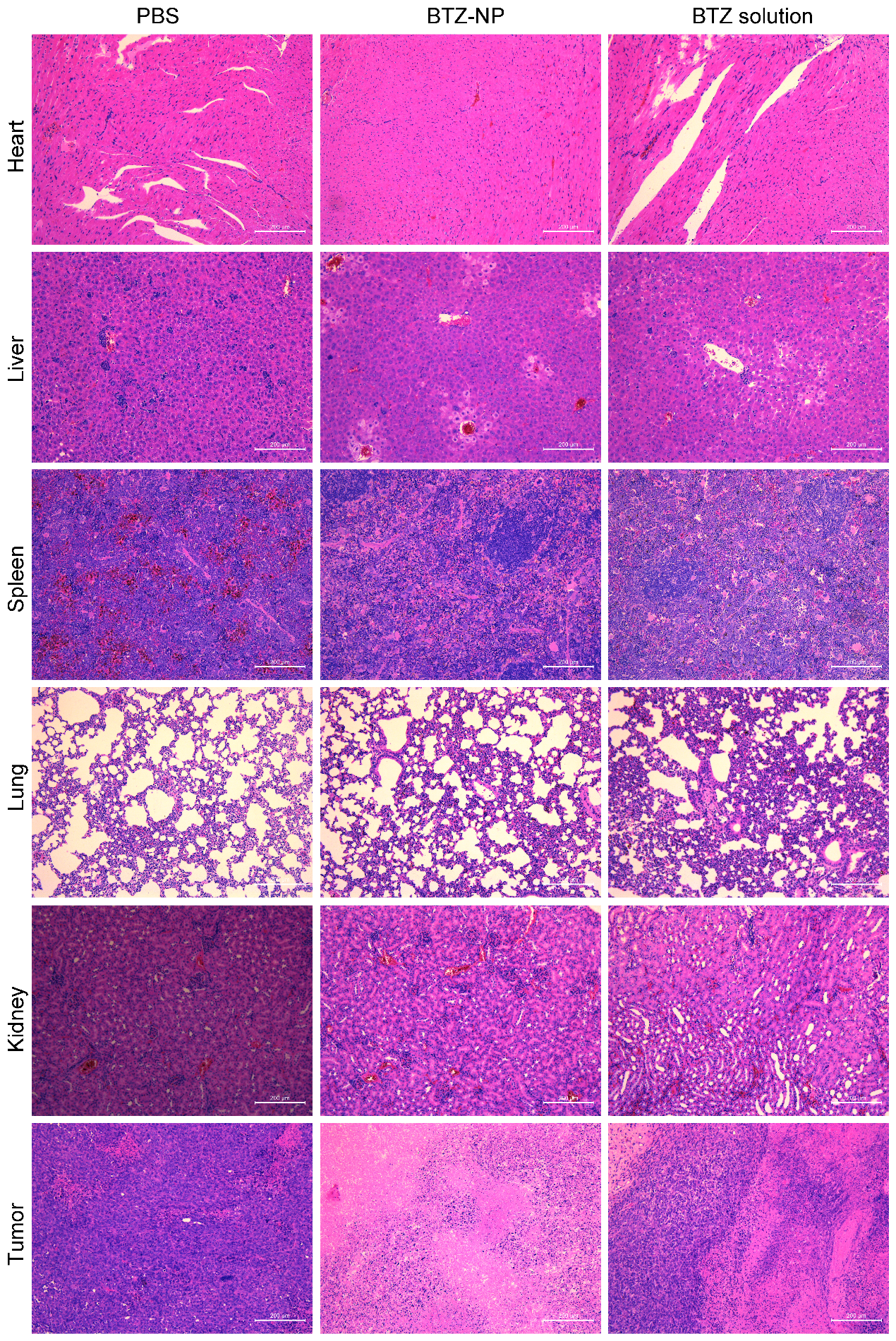


Fig. S3 H & E staining of the tumor tissues and the main organs of heart, liver, spleen, lung, kidney.


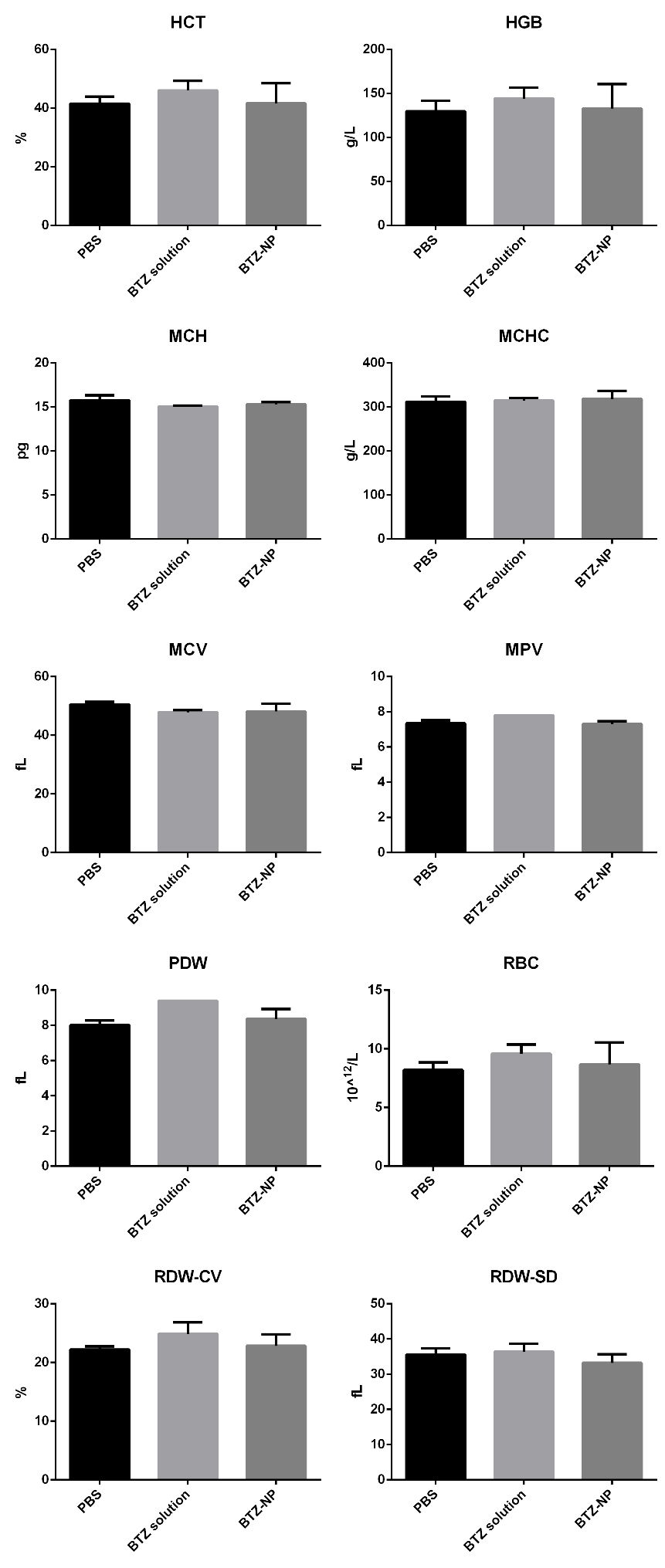


Fig. S4 The blood analysis results of the tumor bearing mice after receiving different treatments.
